# Intraluminal pressure profiles during flexible ureterorenoscopy

**DOI:** 10.1186/s40064-015-1114-4

**Published:** 2015-07-24

**Authors:** Helene Jung, Palle J S Osther

**Affiliations:** Department of Urology, Urological Research Center, Lillebaelt Hospital, University of Southern Denmark, Fredericia, Denmark

## Abstract

**Background:**

Irrigation and instrumentation during ureterorenoscopic procedures may cause increased pressure in the renal pelvis (PP) with potential harmful adverse effects. In order to assess the pressure increases during ureterorenoscopy, we measured the intraluminal renal pelvic pressure during retrograde intrarenal stone surgery (RIRS).

**Methods:**

Twelve patients admitted for RIRS were included. Irrigation rate was standardized to 8 ml/min. A ureteral catheter was retrogradely placed in the renal pelvis for PP measurements. PP was measured one time per second during insertion of the Storz Flex-X2 ureteroscope and during stone treatment.

**Results:**

Baseline PP was mean 10(±4.0) mmHg. During simple ureterorenoscopy, PP was mean 35(±10) mmHg. During stone management the average PP was 54(±18) mmHg and pelvic pressure peaks up to 328 mmHg occurred. In a 5-min standardized period of simple ureterorenoscopy, 83 pressure peaks >50 mmHg were measured in average per patient (range 2–238). Forced irrigation with a 20 ml syringe resulted in pressure peaks up to 288 mmHg.

**Conclusion:**

Very high pelvic pressures are obtained during flexible ureterorenoscopy. Taking into consideration that the threshold for pyelovenous backflow is around 30 mmHg, it is concerning that PPs >300 mmHg are not uncommon during these procedures. Methods to monitor and lower the PP during ureterorenoscopy, therefore, are considered of importance.

## Background

Technological advancements such as miniaturization of scopes, superflexible utensils, improved video imaging, laser settings and irrigation systems have expanded the indications for ureterorenoscopy dramatically (Beiko and Denstedt 2007). Flexible ureterorenoscopy with laser vaporization has appeared to be a promising solution for local nephron-sparing treatment of low-grade smaller-size transitional cell carcinoma of the upper urinary tract (Grasso et al. 2012), and in many challenging stone cases flexible ureterorenoscopy is now by many endourologists considered first-line treatment. Until recently flexible ureterorenoscopy was considered to have a limited role in the treatment of intrarenal calculi larger than 2 cm. An important transition is occurring, however, larger and more complex stone burdens now being increasingly addressed ureterorenoscopically (Cohen et al. 2013).

Traditionally, ureterorenoscopic interventions are considered to be very safe. Serious complications such as urosepsis, bleeding and ureteral lesions do occur, however, and since incidence of complications has been clearly linked to operative time (Geavlete et al. 2006; Schuster et al. 2001; Schoenthaler et al. 2012), the newer trends in flexible ureterorenoscopy dealing with more complex upper urinary tract pathology potentially may lead to higher complication rates.

Complications to flexible ureterorenoscopy may be related to increased intraluminal pressure during the procedures. Previously intraluminal pressures during ureterorenoscopy have been measured in patients drained with a nephrostomy tube for some weeks, which may have affected results. The aim of this study was to review the literature regarding ways to reduce pressure and to examine intraluminal pressure profiles during different phases of therapeutic ureterorenoscopy in patients who were not previously stented or nephrostomy deviated.

## Methods

The local ethical committee improved the study and all patients signed informed consent. Patients were recruited from Department of Urology, Lillebaelt Hospital, University of Southern Denmark, Fredericia, Denmark. Twelve patients admitted for Retrograde Intrarenal Stone Surgery (RIRS) due to stones in the renal calyces or pelvis, were included. None of the patients had JJ-stents or ureteral stones prior to surgery. All patients had sterile urine and normal baseline laboratory studies.

During cystoscopy a guide wire (Sensor^®^, Boston Scientific, Natick, USA) was placed in the renal pelvis via a ureteral catheter under fluoroscopic control. A dual lumen ureteral catheter (COOK Medical Inc, Bloomgton, USA) was then inserted and a second guide wire was placed in the renal pelvis. The dual lumen catheter was removed and a 4-F ureteral catheter was placed over one of the guide wires in the renal pelvis for pelvic pressure (PP) measurements.

The pressure catheter was connected to a transducer (Statham Gould # 4523551, Dunlap, England) placed at the level of the kidney and connected to amplifier and monitor (Medistim CardioMed CM-4008, Oslo, Norway). Perfusion was done through the flexible ureteroscope using a pump (3 M, GRASBY 3500^®^, Watford, UK) to secure a standardized irrigation rate of 8 ml/min. Forced irrigation with a 20 ml syringe was used when necessary to improve vision.

Pelvic pressure measurements were initiated when correct placement of the ureteral catheter in the renal pelvis had been secured by fluoroscopy. Baseline pelvic pressure was measured before the ureteroscope was inserted into the ureter. A Flex-X2^®^ 7.5 F (Karl Storz, Tuttlingen, Germany) flexible ureteroscope was used in all patients using a Sensor^®^ wire as a guide. Ureteral access sheaths were not used and instrumentation was accomplished without dilatation of the ureteral orifice.

### Data collection

In order to make proper comparisons of data three standardized study periods were defined during the procedures.

#### Baseline

Two-minute period prior to ureterorenoscopic instrumentation used for baseline PP measurement.

#### Period 1

Five-minute period of ureterorenoscopy including examination of all renal calyces using saline irrigation 8 ml/min.

#### Therapeutic period

The time interval used for stone disintegration and stone removal using Holmium laser fibre, stone baskets and forced irrigation to secure visualization etc.

PP was measured in one-second intervals yielding 60 measurements/min. The “mean PP” was estimated as a mean of all PP measurements during the given period.

A “pressure peak” was defined as a single PP measurement above 50 mmHg. The “maximum pressure peak” was defined as the highest PP measured throughout the procedure in a given patient.

The bladder was continuously drained via a Ch. 8 silicone catheter.

## Results

Stone size ranged from 3 to 9 mm with a mean of 5.7 ± 2.9 mm. The therapeutic period ranged from 3 to 26 min; mean 9.8 min.

The mean PPs are presented in Table 1 for Baseline, Period 1 and Therapeutic period, respectively.

**Table 1:** Pelvic pressure during ureteroscopy

Patient No	Baseline	Period 1	Therapeutic period	Maximum pressure peak
1	8	40	64	101
2	8	38	51	86
3	10	21	25	83
4	11	30	52	104
5	8	45	59	280
6	13	35	80	328
7	10	18	22	90
8	12	14	42	114
9	14	59	65	233
10	9	53	75	226
11	9	23	37	96
12	8	44	76	115
Mean, patient 1–12	10(±4.0)	35(±10)	54(±18)	155(±83)

mmHg. Mean values.

**Fig. 1:**
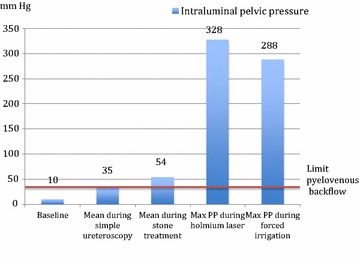
Intraluminal pelvic pressures (PP) observed under baseline conditions and ureterorenoscopy. mmHg.

**Fig. 2:**
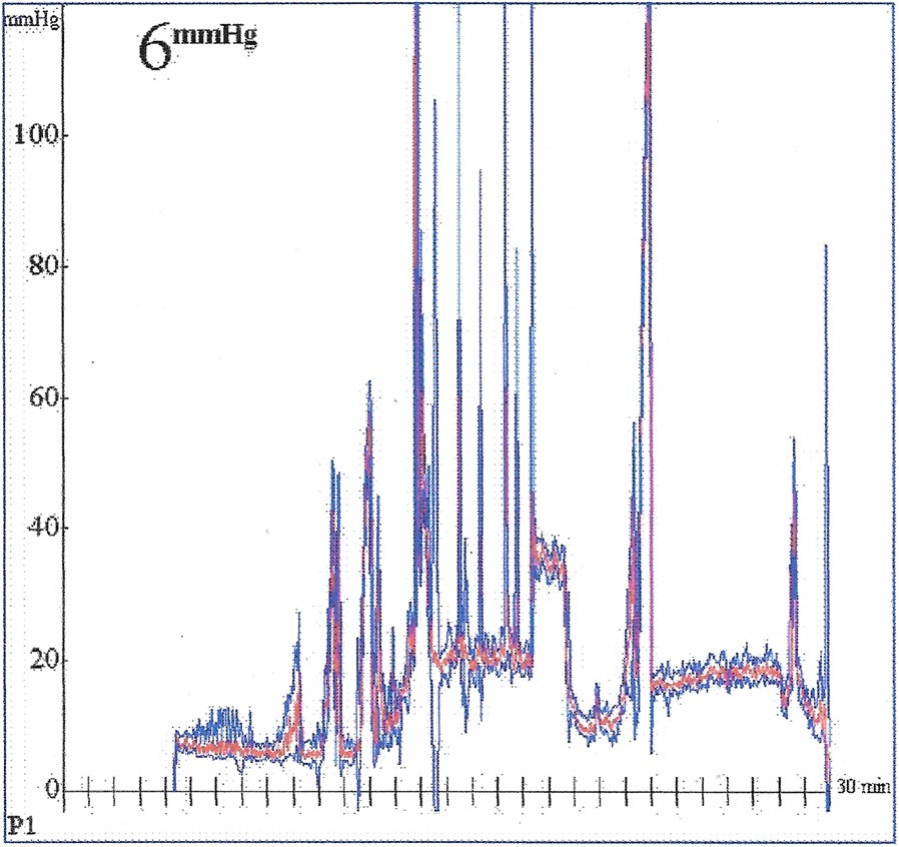
Intraluminal pelvic pressure measurements in a patient during ureterorenoscopy. High pelvic pressures were seen especially during forced irrigation and stone fragmentation (Minutes 14–19).

Baseline PP was mean 10(±4.0) mmHg. During simple ureterorenoscopy (Period 1), PP was mean 35(±10) mmHg. In period 1, 83 pressure peaks >50 mmHg were measured in average per patient (range 2–238) (Figs. 1, 2).

In the therapeutic period (ureterorenoscopy including use of stone basket, Holmium-laser and forced irrigation) the average PP was 54(±18) mmHg and pelvic pressure peaks up to 328 mmHg occurred. The maximum PP obtained in each patient ranged from 83 to 328 mmHg and were seen during the therapeutic period. The average maximum PP in the therapeutic period was 155(±83) mmHg (Fig. 1).

All patients had sterile urine prior to surgery and received Cefuroxime 1.5 g intravenously per-operatively. No intraoperative complications were registered and all patients were discharged within 48 h.

## Discussion

The normal physiological intrarenal pressure is approximately 10 mmHg (Djurhuus 1980) and we demonstrated intraluminal renal pelvic pressures up to 328 mmHg during ureterorenoscopy. The threshold for pyelovenous and pyelosinous back flow has previously been shown to be in the order of 30–45 mmHg (Thomsen 1984). Our study confirms previous reports that this threshold is dramatically exceeded during flexible ureterorenoscopy, which potentially may give rise to septic complications (Wilson 1990). The data highlights the importance of using prophylactic antibiotics in these procedures.

Despite on-going efforts miniaturizing semirigid and flexible ureteroscopes, access difficulties due to tight conditions in the ureter are still to be sufficiently solved. Ureteral dilatation rates of 12–25% are reported in previous series (Rehman et al. 2003) and complications associated with the dilatation procedure have been seen in 6% of cases. Furthermore, ureteral perforation rates of 15% have been observed (Stoller et al. 1992). Using a ureteral access sheath (UAS) Rehman et al. found intrarenal pressures up to 58.9 cm H_2_O during flexible ureteroscopy and recommended the use of UAS to prevent complications caused by pressure elevations (Rehman et al. 2003).

UASs are often used to minimize the hassles of repeated passages of the ureteroscope through the ureter. Auge et al. reported that the mean intrarenal pressure measured through a nephrostomy tube exceeded 94 mmHg when a flexible ureteroscope was present in the renal pelvis. This pressure was reduced to 40 mmHg when a UAS was inserted (Auge et al. 2004). Although the intrapelvic pressure was significantly reduced using the UAS, the pressure levels during ureterorenoscopy still exceeded the level of pyelovenous backflow. Complications associated with the insertion of the UAS were not addressed in this paper. The pressure levels measured in our study in patients who were not previously drained were in the same order of magnitude. Additionally, pressure peaks close to 300 mmHg during forced irrigation and laser fragmentation were demonstrated. It is well known from animal studies and clinical observations that a sustained high pressure results in kidney damage (Jung et al. 2006). Whether these short pressure peaks are harmful for the kidney is still a matter of debate.

In the present study irrigation by gravity supplemented with on-demand flushing using a syringe was used. Using pressure-controlled pumps and other devices for on-demand flushing may have altered the intrarenal pressure profiles. Furthermore, pressure bags may potentially result in sustained high pressures, and usage of such devices is probably not recommendable.

In order to determine the optimal size of an UAS to achieve good irrigation, Ng et al. evaluated the flow rate and the intrarenal pressure during insertion of different access sheaths (Ng et al. 2010). It was concluded that increased UAS diameter did not improve flow when the working channel of the flexible ureteroscope was occupied, but the intrarenal pressure decreased significantly, particularly when using the 16F UAS. Evidence has been put forward, that presence of a UAS can induce transient ureteral ischaemia, promote an acute inflammatory response and give rise to ureteral stricture (Boddy et al. 1989). Lallas et al. found that UAS insertion caused a transient decrease in ureteral blood flow in swine (Lallas et al. 2002). The larger diameter of the sheath, the more pronounced the decrease in blood flow. In a recent study by Traxer and Thomas data on a total of 359 consecutive patients who underwent retrograde intrarenal surgery for kidney stones were prospectively collected at 2 academic centres (Traxer and Thomas 2013). The patients were prospectively evaluated with regard to incidence and severity of ureteral damage due to UAS placement. UAS related ureteral wall lesions were present in 167 patients (46.5%), 13.4% representing high-grade injuries. The authors proposed a classification of UAS related ureteral injuries and showed that JJ pre-stenting significantly decreased the incidence of severe UAS-related damage. However, large prospective trials documenting the rate of stricture formation after the use of UAS are lacking. Abrahams et al. argued against the routine use of UAS due to potential disadvantages such as increased ureteroscopic resistance, increased risk of missing distal ureteral pathology and the fact that the UAS does not necessarily extend all the way to the stone, exposing the most proximal part of the urinary tract to repeated manipulation and risk of perforation and later stricture formation (Abrahams and Stoller 2004). On the other hand, UAS remain the only proven, data driven option to reduce intrarenal pressures during retrograde intrarenal surgery; however their use often mandates the use of a ureteral stent (Rapoport et al. 2007). If a UAS is considered, it seems advisable to use the smallest possible in which the ureteroscope fits, since this will reduce risk of ureteral damage (Traxer and Thomas 2013).

The type and number of different receptors in the upper urinary tract are well documented (Park et al. 2000 ;Malin et al. 1970; Jung et al. 2006). β-adrenergic stimulation causes relaxation of the ureter, while α-adrenergic stimulation causes contraction. Human studies in this area are quite sparse, but we have in earlier studies demonstrated that it was possible to lower the PP during ureterorenoscopy using topical administration of a β-adrenergic agonist (isoproterenol) in the ureter (Jung et al. 2008a, b). No cardiovascular side effects were observed. The trend of extensive pressure rises significantly decreased during isoprenalin irrigation, and the average pressure remained below the level of intrarenal back flow. Similar results were obtained in an experimental swine study showing that isoprenalin significantly reduced the pressure-flow relation during semirigid ureterorenoscopy (Jakobsen et al. 2010).

We have previously shown that intrarenal pressure correlates to pain (Pedersen et al. 2012). Unfortunately, postoperative pain was not monitored in our series. Reducing intrarenal pressures during ureterorenoscopy may, however, also have important implications with regard to pain, and should be addressed in future research.

### Limitations: retrograde pressure measurement

The pelvic pressure measurements in the present study were obtained through a retrogradely inserted pressure transducer. Endoluminal devices might themselves alter ureteral peristalsis and hence may not optimally be applied for measurement of peristaltic activity and intraluminal pressure. However, the baseline pelvic pressures measured in this study were similar to pelvic pressures observed in previous studies using antegradely inserted catheters for pressure measurement (Wilson 1990; Auge et al. 2004; Jakobsen et al. 2007; Schwalb et al. 1993). The comparable pelvic pressure levels despite the different techniques employed serve as validation of the method applied in this study. Ethical acceptable alternatives for pelvic pressure measurement during ureteroscopy in humans are not available.

## Conclusion

The intraluminal renal pressure increased dramatically during therapeutic ureterorenoscopy. Intraluminal pressure profiles measured retrogradely confirmed results of previous studies using antegrade pressure measurements. Pressure peaks >300 mmHg were detected. This may have important implications for both septic complications and postoperative pain. Access sheaths may reduce the pressure, but may also imply risks and complications in terms of ureteral damage and subsequent stricture formation. Devices for monitoring intraluminal pressure during the procedures would be desirable. Finally, pharmacological manipulation of ureteral tone and intraluminal pressure may be of potential value during ureterorenoscopy to prevent complications and facilitate access.

## References

[CR1] Abrahams HM, Stoller ML (2004). The argument against the routine use of ureteral access sheaths. Urol Clin North Am.

[CR2] Auge BK, Pietrow PK, Lallas CD, Raj GV, Santa-Cruz RW, Preminger GM (2004). Ureteral access sheath provides protection against elevated renal pressures during routine flexible ureteroscopic stone manipulation. J Endourol Endourol Soc.

[CR3] Beiko DT, Denstedt JD (2007). Advances in ureterorenoscopy. Urol Clin North Am.

[CR4] Boddy SA, Nimmon CC, Jones S, Ramsay JW, Britton KE, Levison DA (1989). Irrigation and acute ureteric dilatation—as for ureteroscopy. Br J Urol.

[CR5] Cohen J, Cohen S, Grasso M (2013). Ureteropyeloscopic treatment of large, complex intrarenal and proximal ureteral calculi. BJU Int.

[CR6] Djurhuus JC (1980). Aspects of Renal Pelvic Function (Thesis).

[CR7] Geavlete P, Georgescu D, Nita G, Mirciulescu V, Cauni V (2006). Complications of 2735 retrograde semirigid ureteroscopy procedures: a single-center experience. J Endourol Endourol Soc.

[CR8] Grasso M, Fishman AI, Cohen J, Alexander B (2012). Ureteroscopic and extirpative treatment of upper urinary tract urothelial carcinoma: a 15-year comprehensive review of 160 consecutive patients. BJU Int.

[CR9] Jakobsen JS, Holst U, Jakobsen P, Steen W, Mortensen J (2007). Local and systemic effects of endoluminal pelvic perfusion of isoproterenol: a dose response investigation in pigs. J Urol.

[CR10] Jakobsen JS, Jung HU, Gramsbergen JB, Osther PJ, Walter S (2010). Endoluminal isoproterenol reduces renal pelvic pressure during semirigid ureterorenoscopy: a porcine model. BJU Int.

[CR11] Jung HU, Frimodt-Moller PC, Osther PJ, Mortensen J (2006). Pharmacological effect on pyeloureteric dynamics with a clinical perspective: a review of the literature. Urol Res.

[CR12] Jung H, Norby B, Frimodt-Moller PC, Osther PJ (2008). Endoluminal isoproterenol irrigation decreases renal pelvic pressure during flexible ureterorenoscopy: a clinical randomized, controlled study. Eur Urol.

[CR13] Jung HU, Jakobsen JS, Mortensen J, Osther PJ, Djurhuus JC (2008). Irrigation with isoproterenol diminishes increases in pelvic pressure without side-effects during ureterorenoscopy: a randomized controlled study in a porcine model. Scand J Urol Nephrol.

[CR14] Lallas CD, Auge BK, Raj GV, Santa-Cruz R, Madden JF, Preminger GM (2002). Laser Doppler flowmetric determination of ureteral blood flow after ureteral access sheath placement. J Endourol Endourol Soc.

[CR15] Malin JM, Deane RF, Boyarsky S (1970). Characterisation of adrenergic receptors in human ureter. Br J Urol.

[CR16] Ng YH, Somani BK, Dennison A, Kata SG, Nabi G, Brown S (2010). Irrigant flow and intrarenal pressure during flexible ureteroscopy: the effect of different access sheaths, working channel instruments, and hydrostatic pressure. J Endourol Endourol Soc.

[CR17] Park YC, Tomiyama Y, Hayakawa K, Akahane M, Ajisawa Y, Miyatake R (2000). Existence of a beta3-adrenoceptro and its functional role in the human ureter. J Urol.

[CR18] Pedersen KV, Liao D, Osther SS, Drewes AM, Gregersen H, Osther PJ (2012). Distension of the renal pelvis in kidney stone patients: sensory and biomechanical responses. Urol Res.

[CR19] Rapoport D, Perks AE, Teichman JM (2007). Ureteral access sheath use and stenting in ureteroscopy: effect on unplanned emergency room visits and cost. J Endourol.

[CR20] Rehman J, Monga M, Landman J, Lee DI, Felfela T, Conradie MC (2003). Characterization of intrapelvic pressure during ureteropyeloscopy with ureteral access sheaths. Urology.

[CR21] Schoenthaler M, Wilhelm K, Kuehhas FE, Farin E, Bach C, Buchholz N (2012). Postureteroscopic lesion scale: a new management modified organ injury scale—evaluation in 435 ureteroscopic patients. J Endourol Endourol Soc.

[CR22] Schuster TG, Hollenbeck BK, Faerber GJ, Wolf JS (2001). Complications of ureteroscopy: analysis of predictive factors. J Urol.

[CR23] Schwalb DM, Eshghi M, Davidian M, Franco I (1993). Morphological and physiological changes in the urinary tract associated with ureteral dilation and ureteropyeloscopy: an experimental study. J Urol.

[CR24] Stoller ML, Wolf JS, Hofmann R, Marc B (1992). Ureteroscopy without routine balloon dilation: an outcome assessment. J Urol.

[CR25] Thomsen HS (1984). Pyelorenal backflow. Clinical and experimental investigations. Radiologic, nuclear, medical and pathoanatomic studies. Dan Med Bull.

[CR26] Traxer O, Thomas A (2013). Prospective evaluation and classification of ureteral wall injuries resulting from insertion of a ureteral access sheath during retrograde intrarenal surgery. J Urol.

[CR27] Wilson WTPG (1990). Intrarenal pressures generated during flexible deflectable ureterorenoscopy. J Endourol Endourol Soc.

